# Chemokines as regulators of CAR-T cell trafficking, function, and context-dependent tumor cell death

**DOI:** 10.3389/fimmu.2026.1963794

**Published:** 2026-10-07

**Authors:** Ksenia Levchuk, Alexey Petukhov, Shynggys Tursymbek, Kuanysh Seitkamal, Arailym Bertleuova, Denis Bulanin, Nikolai A. Barlev

**Affiliations:** 1 Institute of Cytology, Russian Academy of Sciences, St. Petersburg, Russia; 2 Laboratory of Molecular Oncology, National Laboratory Astana, Astana, Kazakhstan; 3 Department of Biomedical Sciences, School of Medicine, Nazarbayev University, Astana, Kazakhstan; 4 Limited Liability Partnership (LLP) Center of Hematology, Astana, Kazakhstan

**Keywords:** CAR-T cells, chemokines, functional chemokine gradients, immune resistance, immunogenic cell death, solid tumors, tumor microenvironment

## Abstract

Chimeric antigen receptor (CAR)-T cell therapy has transformed the treatment of hematological malignancies, yet its efficacy in solid tumors remains limited by inefficient trafficking, suppressive myeloid and regulatory T-cell populations, and metabolic stress. These barriers contribute to incomplete tumor cell killing and loss of CAR-T cell function. Chemokines are usually viewed as migratory guides, but in solid tumors they also orchestrate immune cell positioning and inflammatory state. In this review, we discuss how chemokine gradients form inflammatory niches and control CAR-T-cell recruitment, intratumoral distribution, retention, and egress. We also examine how chemokine programs interact with cytokine signaling and regulated tumor cell death to shape local and potentially tissue-wide antitumor immunity, including through the release of damage-associated molecular patterns (DAMPs). Finally, we outline translational strategies that include engineered chemokines and matched chemokine receptor programs to enhance the efficacy of chemokine/cytokine-armored CAR-T cells, and related combination therapies.

## Graphical Abstract

**Figure:**
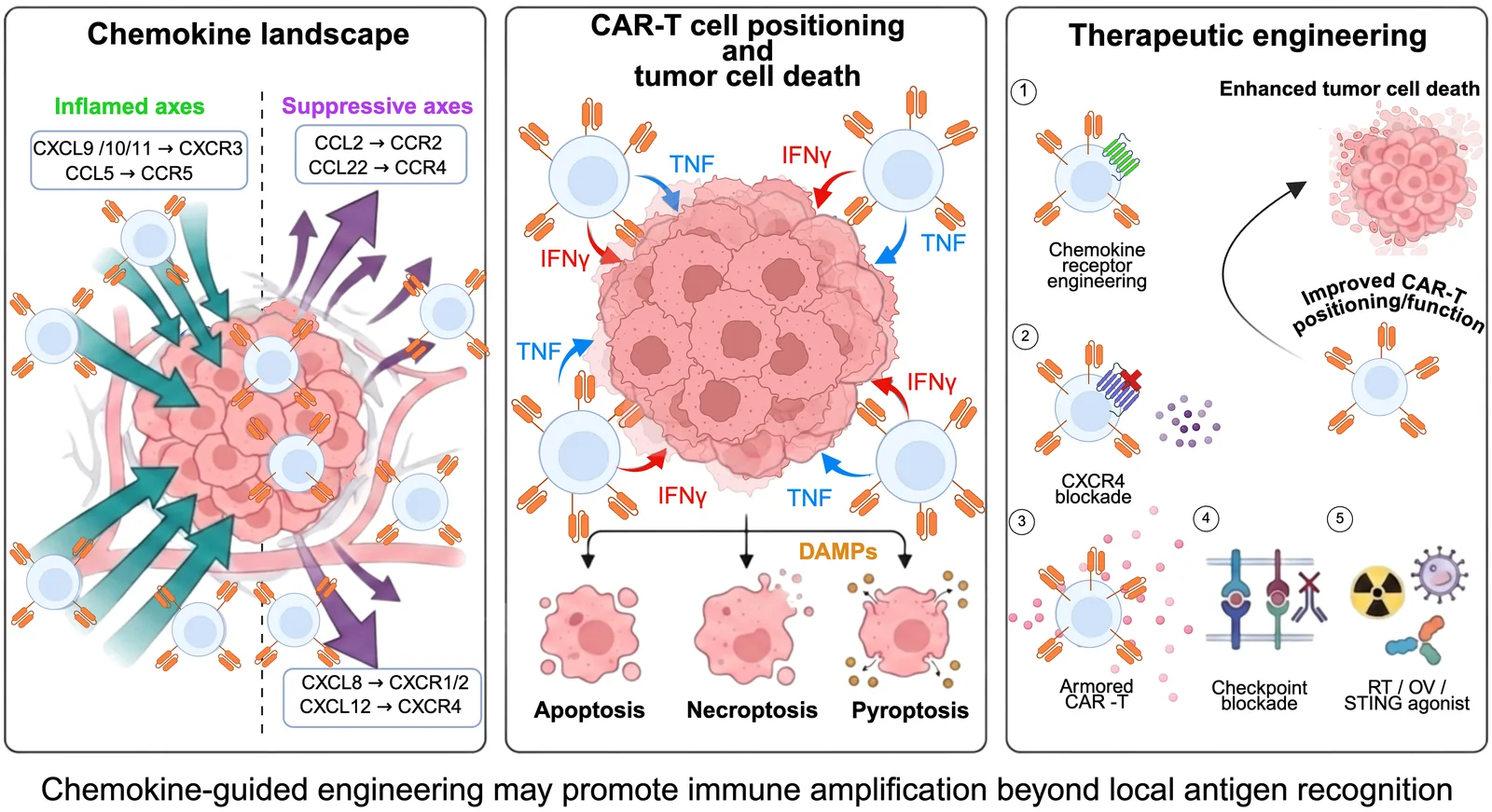
Schematic overview of context-dependent chemokine programs and investigational CAR-T engineering strategies. Apoptosis, necroptosis, and pyroptosis are alternative outcomes determined by the available tumor cell death machinery. Tissue-wide immune amplification serves as a proposed consequence. DAMPs, damage-associated molecular patterns; OV, oncolytic virus; RT, radiotherapy; STING, stimulator of interferon genes.

## 1 Introduction

CAR-T cell therapy has revolutionized the treatment of several B-cell malignancies (1, 2). According to the results of successful clinical trials, genetically engineered T-cells can induce deep and durable remissions in these diseases (1, 2). By contrast, the efficacy of CAR-T-cell therapy in solid tumors remains limited (1–4). Several factors may account for this dramatic difference. For example, deposited extracellular matrix (ECM) proteins can physically restrict T-cell entry into solid tumors (5, 6). In addition, myeloid-derived suppressor cells (MDSCs) and regulatory T-cells that accumulate in the lesion can restrain CAR-T cell cytotoxicity (3, 7, 8). Furthermore, persistent inflammatory signaling from tumor-associated macrophages (TAMs) may weaken CAR-T cell function further. Finally, unfavorable physicochemical conditions, including hypoxia, acidosis, and lactate accumulation, impose metabolic stress on T-cells and promote their early dysfunction (9–11). Together, these factors reduce CAR-T cell accumulation and activity in solid tumors. A local response may still develop, yet it is less likely to spread across the tissue and achieve durable tumor clearance.

Chemokines are small secreted proteins classified into the CC, CXC, CX3C, and XC subfamilies according to the number and spacing of conserved N-terminal cysteine residues (12). Most chemokine receptors are seven-transmembrane G-protein-coupled receptors, and receptor preference is determined by the ligand sequence and three-dimensional interaction rather than by cysteine spacing alone (13, 14). Ligand binding changes receptor conformation and activates heterotrimeric G proteins. Subsequent signaling through Gα and Gβγ then engages PLC-PKC, MAPK, phosphoinositide-3-kinase, and actin-remodeling pathways (14–16). These coordinated events support polarization, adhesion, and migration along a chemokine gradient (14–16). Chemokines therefore function as major regulators of leukocyte trafficking and, together with cytokines, mediate communication among malignant, stromal, and immune cells (12, 17, 18). In tumors, chemokine receptor networks influence immune cell recruitment, spatial positioning, and functional state and can consequently support either antitumor immunity or immune suppression (17–20).

Accordingly, a growing body of published evidence suggests that the efficacy of immunotherapy is critically affected by the chemokine milieu within the tumor microenvironment (21, 22). In particular, effective CAR-T-cell therapy appears to require not only successful antigen recognition but also a favorable inflammatory microenvironment capable of supporting and sustaining the antitumor response at the tissue level (23). Cytokines secreted by CAR-T cells diffuse beyond the original immune synapse. Exposure of malignant and stromal cells to these cytokines can shift the local chemokine output. Endogenous immune cell populations may then enter the lesion, carrying inflammation beyond the site at which the CAR was engaged. Within this local response, TNF- and IFNγ-driven cytokine signaling can engage broader inflammatory programs involving activation of the NF-kB-dependent transcription program and, in some cellular contexts, inflammasome pathways (24–28). Importantly, these effects extend beyond immune cell recruitment, because cytokine exposure can also alter tumor cell antigen presentation, death receptor signaling, and susceptibility to immune-mediated killing (27–29).

CAR-T-cell-mediated elimination of tumor cells may involve pyroptotic, apoptotic, or necroptotic cell death pathways. In inflammatory models outside CAR-T therapy, shared death-signaling components can assemble into PANoptosomes and initiate PANoptosis (30). The engagement of these pathways depends on the presence of intact death-signaling machinery in the target cells and can result in the release or surface exposure of antigens and danger-associated molecular patterns (DAMPs), each with distinct immunogenic consequences (31–33). DAMPs released or exposed by dying tumor cells can activate innate immune sensing, while chemokines produced within the same microenvironment can recruit and spatially organize responsive immune populations (18, 31, 32). In some lesions, these effects may remain confined to the immediate CAR-T-cell-target-cell interface; in others, DAMP-dependent antigen presentation and chemokine-guided recruitment may promote activation of endogenous tumor-reactive T-cells and NK cells beyond the initially engaged CAR-T-cell population (18, 32–35).

The same circuitry can also favor resistance. With chronic inflammation, the lesion becomes enriched in suppressive macrophages, MDSCs, and regulatory T-cells. Once established, these populations feed back on the niche through further cytokine and chemokine production. Sustained IFNγ- and NF-kB-dependent signaling may also activate adaptive-resistance programs and contribute to CAR-T cell dysfunction. For example, prolonged chemokine signaling can help stabilize suppressive stromal and myeloid niches in which NF-kB remains active and T-cells are dysfunctional. Within tumor cells, NF-kB directly induces c-FLIP and BCL-XL, making them less susceptible to inflammatory cell death (24–26). The combined action of these pathways influences whether immune activity is restricted to scattered foci or develops into a coordinated response across the tissue.

Tumor-specific defects in apoptotic or necroptotic pathways may further distort the action of inflammatory cytokines, protecting cancer cells from effective cell death despite the continuous immune pressure (24–26, 29, 31, 33).

In this review, we discuss how chemokines shape the integrated tumor response to CAR-T cell immunotherapy by regulating T-cell recruitment, spatial organization, inflammatory signaling, and tumor cell death. We examine their interplay with cytokine TNF/IFNγ signaling, regulated cell death pathways, and the suppressive tumor microenvironment. We then evaluate engineered chemokines, matched chemokine receptor programs, armored CAR-T cells, microenvironmental combinations, safety controls, and single-cell and spatial strategies for patient selection and treatment monitoring.

## 2 Chemokines as protumor and antitumor immunity regulators

The effectiveness of immunotherapy depends in part on the intensity and spatial pattern of immune cell infiltration. Chemokines are central to communication between malignant and immune cells and help distinguish T-cell-inflamed (“hot”) from non-T-cell-inflamed or immune-excluded (“cold”) tumors (17, 18, 36). “Hot” tumors are characterized by an inflammatory tumor microenvironment. In some tumors, a high mutational burden contributes to immunogenicity by generating neoantigens that can be recognized by tumor-reactive T-cells (18, 36). Following antigen recognition, intratumoral CD8^+^ T-cells can release IFNγ and TNF. Malignant and stromal cells exposed to these cytokines increase chemokine production, forming gradients that attract cytotoxic T-lymphocytes, NK-cells, and dendritic cells (17, 18). In turn, recruited cells can reinforce local inflammation and antigen presentation.

By contrast, “cold” tumors contain few intratumoral T-cells or show an immune-excluded pattern in which lymphocytes remain at the invasive margin. Low neoantigenicity is one possible cause, but weak interferon signaling and deficient expression of T-cell-recruiting chemokines can also prevent infiltration (37). Even after entry, CD8^+^ T-cells may become trapped in prolonged contacts within tumor dendritic-cell networks (38) or migrate along stromal fibers without reaching tumor cell nests (20). Such tumors may also preferentially recruit MDSCs, TAMs, and regulatory T-cells through CCL2, CXCL8, CXCL12, or CCL22 (17, 20, 39). These contrasting spatial arrangements are summarized in **Figure 1**.

**Figure 1:**
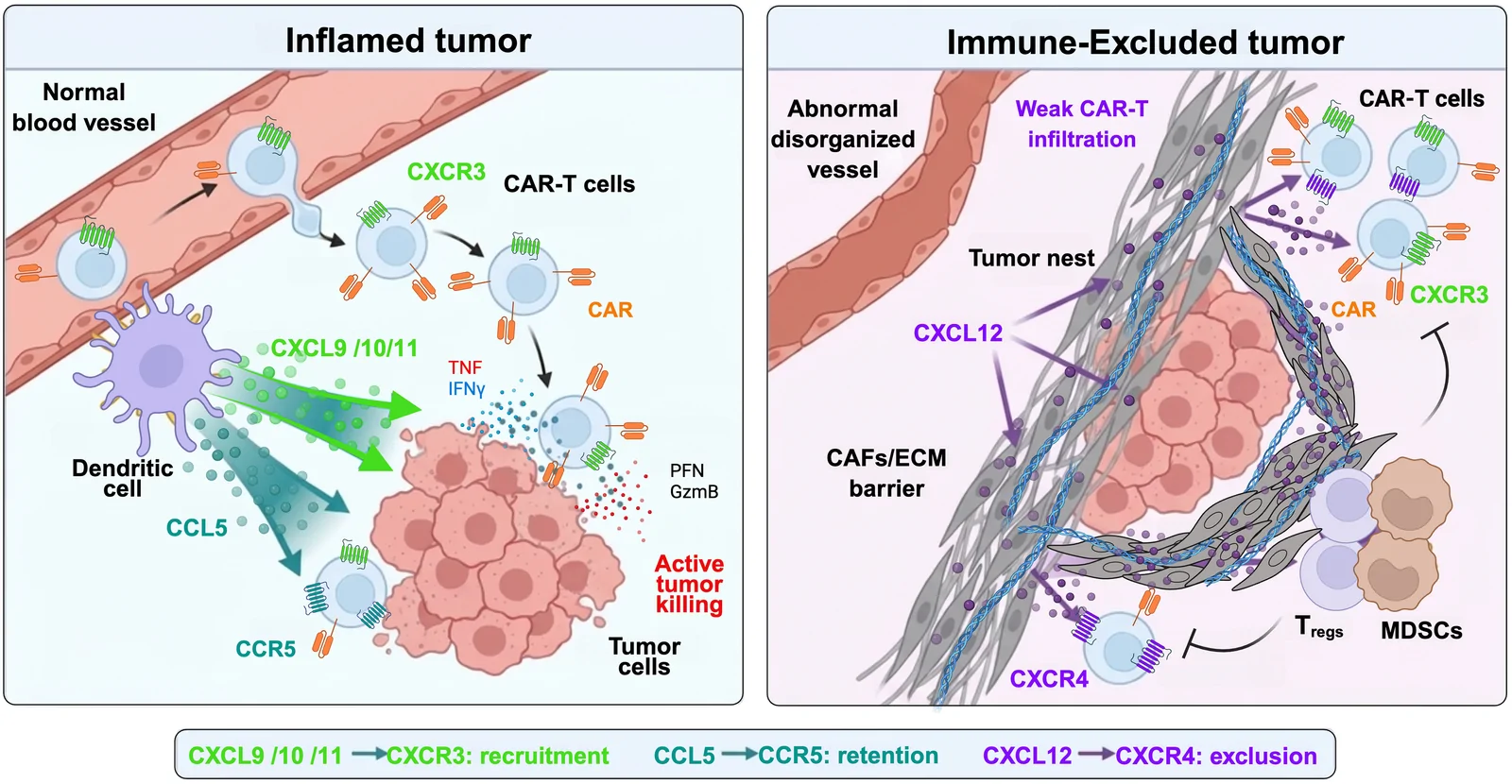
Chemokine-guided CAR-T cell positioning in inflamed and immune-excluded tumors. In inflamed tumors, the CXCL9/CXCL10/CXCL11-CXCR3 axis supports CAR-T cell recruitment across permissive or activated tumor endothelium. In selected contexts, CCL5-CCR5 signaling may support recruitment, engraftment, or local positioning within the lesion, where recognition of tumor antigen is followed by release of TNF, IFNγ, perforin, and granzyme B. Immune-excluded tumors show a different arrangement: disorganized vasculature and a CAF/ECM-rich barrier impede infiltration, CAF-derived CXCL12 can spatially sequester CXCR4-positive CAR-T cells in the stromal compartment in selected tumor models, and Tregs and MDSCs reinforce exclusion. CAFs, cancer-associated fibroblasts; ECM, extracellular matrix; MDSCs, myeloid-derived suppressor cells; Tregs, regulatory T-cells.

### 2.1 Chemokine programs associated with antitumor immunity

Among chemokine programs associated with antitumor immunity, the CXCL9/CXCL10/CXCL11-CXCR3 axis is of particular importance (19, 34, 40, 41). Their expression is induced by IFNγ and TNF after immune cells encounter their targets. Activated CD8^+^ T-cells and NK-cells express CXCR3, the receptor shared by these ligands. Across several solid tumor types, lesions rich in CXCL9 and CXCL10 tend to contain more T-cells and to respond better to immunotherapy (34, 42). Interruption of CXCR3 signaling, by contrast, impairs effector T-cell trafficking and can reduce the efficacy of adoptive T-cell therapy or checkpoint blockade (19).

This axis is nevertheless context-dependent. In melanoma, CXCL10 has also been linked to angiogenesis and growth of CXCR3-expressing tumor cells (43).

CXCL16 exists in both a transmembrane form, which functions as an adhesion/scavenger molecule, and a soluble chemokine form generated by proteolytic shedding. Membrane-bound CXCL16 can promote adhesion of CXCR6^+^ lymphocytes to CXCL16-expressing cells, whereas soluble CXCL16 acts as a chemoattractant for activated T-cells. IFNγ and TNF can increase *CXCL16* expression in vascular and stromal cells, while ADAM10 mediates its constitutive shedding from the cell surface (44). In pancreatic tumor models, enforced *CXCR6* expression enhanced the accumulation and antitumor activity of tumor-specific T-cells within *CXCL16*-expressing lesions (45). In a separate study, surface CXCL16 on tumor-associated myeloid cells may promote CXCR6-dependent T-cell-myeloid-cell interactions and, in a glioblastoma model, was associated with T-cell dysfunction (46). Thus, the biological effects of the CXCL16-CXCR6 axis depend not only on ligand abundance but also on CXCL16 proteoform, cellular source, shedding, and spatial distribution.

Chemokines CCL3, CCL4, and CCL5 are produced by activated immune cells and tumor-associated stromal cells during inflammation and are part of the “hot tumor” chemokine signature (18). CCL5 can promote the recruitment of cytotoxic T-cells and, together with CXCL9, is associated with response to checkpoint blockade (47). However, CCR5 signaling is context-dependent: depending on the tumor type, spatial niche, and associated cytokines, CCR5 ligands may either recruit NK-cells and cytotoxic lymphocytes (48), or conversely, enrich the tumor with suppressor immune cells (49). This duality of chemokine action highlights the functional complexity and context dependence of tumor-chemokine molecular networks (50).

Experimentally supplying an immune-excluded tumor with effector-recruiting chemokines can draw cytotoxic cells into the tissue. In a three-dimensional collagen model, local chemokine production promoted cytotoxic T-lymphocyte accumulation. Following recognition through the endogenous T-cell receptor, murine cytotoxic T-lymphocytes secreted CCL3 and CCL4, supporting recruitment of additional cytotoxic T-lymphocytes; analogous swarming behavior was observed with human CAR-T cells (35).

### 2.2 Chemokine programs associated with protumor immunity

In resistant or immune-excluded tumors, CCL2, CCL22, CXCL8, and CXCL12 can favor recruitment or retention of suppressive myeloid and regulatory populations, thereby limiting CAR-T cell function and persistence (17, 20, 39). These chemokines are not intrinsically immunosuppressive: their effects depend on cellular source, concentration, timing, tissue localization, and the populations that express the corresponding receptors.

CCL2 is a prominent component of immunosuppressive tumor microenvironments. CCL2, through CCR2 signaling, promotes recruitment of monocytes, which can contribute to myeloid-derived suppressor cell (MDSC) and tumor-associated macrophage (TAM) populations that suppress T-cell function (51–54). Tumor cell-intrinsic signaling can also upregulate CCL2 and thereby contribute to a protumoral microenvironment (51, 52).

CCL2 activity is also altered post-translationally: its nitration in tumor tissue reduces chemotaxis of antigen-specific T-cells while retaining myeloid-cell chemotactic activity at high concentrations (55). Arginase, reactive oxygen species, IL-10, TGFβ, and checkpoint ligands produced or expressed by these myeloid populations suppress cytotoxic T-lymphocyte activity. A CCL2-rich tumor can therefore remain inflamed without becoming vulnerable to immune attack.

CXCL8 (IL-8) signaling through CXCR1 and CXCR2 receptors can promote neutrophil and MDSC recruitment, angiogenesis, and stromal remodeling. Furthermore, elevated CXCL8 is frequently associated with an immunosuppressive tumor microenvironment (56, 57). CXCL8 is one of several ELR^+^ CXC chemokines recognized by CXCR2; other ligands include CXCL1, CXCL2, CXCL3, CXCL5, and CXCL6 (58). Importantly, although this signaling axis is generally associated with tumor-promoting effects, its exact functional consequences depend on the specific ligand and tissue context (56, 59). CXCR2 is expressed not only by neutrophils but also by mast cells, monocytes, macrophages, and selected endothelial and epithelial cell populations (58, 59). High CXCL8 abundance has been associated with neutrophil-rich infiltrates and reduced clinical benefit from immune checkpoint blockade (57, 60, 61). CCL22 can organize regulatory T-cell interactions with dendritic cells in lymph nodes (62). In tumor settings, the CCL22-CCR4 axis can recruit regulatory T-cells and may thereby indirectly impair CAR-T-cell persistence and function (39, 63).

In several tumor models, cancer-associated fibroblasts (CAFs) are prominent sources of CXCL12. Peritumorally concentrated CXCL12 can restrict T-cell infiltration through stromal sequestration and immune repulsion, whereas appropriately organized CXCL12 gradients can promote the recruitment of CXCR4^+^ effector cells. Thus, the CXCL12-CXCR4 axis may either oppose or support antitumor immunity depending on ligand concentration and spatial organization (20, 56, 64, 65). T-cell trafficking also depends on mechanisms governing tissue egress. Tissue-resident memory T-cells downregulate S1PR1, thereby reducing their responsiveness to sphingosine-1-phosphate-mediated egress signals and promoting local retention (66). The CXCL12-CXCR4 axis can likewise regulate both tissue entry and exit. In a preclinical melanoma model, CXCL12 produced by tumor-associated lymphatic vessels positioned CXCR4^+^ CD8^+^ T-cells at the tumor periphery and promoted their lymphatic egress; inhibition of CXCR4 or loss of lymphatic CXCL12 increased T-cell retention and improved tumor control (67). Accordingly, the biological consequences of CXCL12-CXCR4 signaling cannot be inferred from ligand abundance alone but instead reflect the balance among recruitment, retention, immune repulsion, and egress.

## 3 Oncogenic control of chemokine expression and tumor microenvironment composition

The tissue from which a tumor arises leaves an imprint on its chemokine and cytokine profile and, consequently, on the local immune microenvironment. Much of the variation among tumors, however, is generated by transcriptional programs downstream of oncogenic signaling. Activating KRAS substitutions, usually at G12, G13, or Q61, can sustain PI3K-AKT, MAPK-ERK, and NF-kB signaling and thereby connect tumor growth with inflammation and remodeling of the immune environment (68).

Additional genetic lesions are common in KRAS-driven tumors, with *TP53* among the genes frequently affected. In response to oncogenic stress, wild-type p53 undergoes multiple post-translational modifications (69, 70) that can promote senescence and cell death, thereby constraining malignant transformation (71, 72). Human EHMT2/G9a can also enhance p53-dependent PUMA transcription through a methylation-independent mechanism involving increased histone acetylation at the PUMA promoter (73). Conversely, loss of p53 activity through enhanced degradation (74) or mutational inactivation of the *TP53* locus removes this tumor-suppressive restraint (75). The consequences of *TP53* mutation are not necessarily limited to loss of p53 function. Certain mutations, particularly within the DNA-binding domain, can confer gain-of-function properties that promote invasion, tumor progression, and other malignant phenotypes (76).

Inflammatory chemokine regulation also varies with p53 status. In ovarian cancer cells, restoration of wild-type p53 reduced TNF-induced expression of *CXCL1, CXCL2, CXCL3*, and *CXCL8* by attenuating NF-kB signaling (77). IκB became more stable after p53 restoration, apparently because its proteasomal turnover was reduced, and NF-kB activation in response to TNF declined (77). Conversely, mutant *TP53* in serous ovarian tumors was associated with higher *CCL8, CCL20, CXCL10*, and *CXCL11* expression. The immune consequences of this inflammatory expression profile cannot be inferred from chemokine abundance alone (78).

Additional oncogenic lesions can intensify the inflammatory output of KRAS. NF-kB signaling is required for tumor development in a *KRAS*-driven lung cancer model (79), while KRAS-MYC cooperation provides another route to aggressive disease (80). One illustration comes from colorectal cancer, where KRAS(G12D) increases CXCL3 and thereby favors the influx of CXCR2^+^ MDSCs (81). Murine lung tumors with mutant KRAS likewise produce several CXCR2 ligands - CXCL1 (KC), CXCL2 (MIP-2), and CXCL5 (LIX) - that favor myeloid-cell accumulation (82). In a KRAS(G12D) lung adenoma model, activation of MYC converted relatively indolent lesions into rapidly proliferating inflammatory adenocarcinomas. In the same model, CCL9 and IL-23 induction altered the composition of the infiltrate: suppressive macrophages entered the tumors, whereas B-cells, cytotoxic T-lymphocytes, and NK-cells were excluded from the microenvironment (83).

Taken together, tumor genotype contributes to the composition of the immune infiltrate. Alterations in *KRAS, TP53, MYC*, and other drivers can change chemokine production, biasing recruitment toward effector cells in some tumors and suppressive populations in others. These shifts influence infiltration, immune escape, and treatment response. Whether mutation status and chemokine measurements together can predict CAR-T cell activity well enough to guide patient selection remains unknown.

## 4 Chemokine regulation of CAR-T cell recruitment, retention, and spatial distribution

Inefficient trafficking and inadequate intratumoral distribution of CAR-T cells remain major obstacles to effective immunotherapy in solid tumors. Sequential chemokine signal relays illustrate why cellular source and timing matter. NK-cell-derived CCL5 and XCL1 recruit conventional type 1 dendritic cells (cDC1) (84), and tumor-resident Batf3-dependent dendritic cells are required for efficient trafficking of cytotoxic T-lymphocytes and for adoptive T-cell therapy in preclinical models (85). Thus, the immune population that arrives first can establish signals needed for recruitment and positioning of later effector populations. In a solid tumor, however, these events unfold within heterogeneous tissue containing dense stroma, abnormal vessels, and physically separated inflammatory niches. These features restrict both the number of cytotoxic lymphocytes that accumulate and how far they disperse, so CAR-T cells often fail to attain the tissue-wide coverage required for durable tumor control (4).

Atypical chemokine receptors (ACKRs) also shape the tumor chemokine landscape by regulating local ligand availability and distribution (86, 87). ACKR2, ACKR3, and ACKR4 scavenge their cognate chemokines, whereas endothelial ACKR1 transports chemokines to the luminal surface and retains them for presentation to circulating leukocytes (86). These activities influence chemokine gradients and immune-cell recruitment in a manner that depends on receptor identity and anatomical location (86, 87). For CAR-T-cell trafficking, this suggests that chemokine production alone may not predict the gradients encountered during vascular entry or intratumoral migration; local ACKR activity may modify the chemokine cues available to infiltrating CAR-T cells.

### 4.1 Chemokine mismatch and insufficient penetration

A major obstacle to recruitment is mismatch between chemokines produced in the tumor and receptors retained by the infused CAR-T cells. Solid tumors can express inflammatory chemokines in response to IFNγ and TNF (18, 20, 34), but these ligands may be poorly matched to the receptor repertoire of activated T-cells. Ovarian cancer, pancreatic cancer, mesothelioma, and glioblastoma can produce CCL2 or CXCL8 (88–90), whereas conventional CAR-T cells may express insufficient CCR2, CXCR1, or CXCR2 to follow these gradients efficiently (1, 88–90). CAR-T cells may consequently lose the trafficking competition to CCR2- or CXCR1/2-expressing suppressive myeloid populations despite preserved antigen recognition. Thus, engineering CAR-T cells to express CCR2b, CXCR1, or CXCR2 can improve migration, intratumoral accumulation, and antitumor activity in preclinical models (89, 90).

The CXCL9/CXCL10/CXCL11-CXCR3 axis illustrates a common form of chemokine mismatch. In many tumors, the limiting component is inadequate ligand availability rather than absence of CXCR3 on activated T-cells. Low-grade gliomas with poor CD8^+^ T-cell infiltration show reduced CXCL9 and CXCL10; DNA methylation and histone modifications can silence the expression of TH1-type chemokines. Furthermore, IFNγ-driven immunoediting can select tumor variants with reduced CXCL9 production (20). The IFNγ signaling that induces these ligands may itself remain spatially confined (91). Strategies that restore local ligand expression or improve CXCR3-dependent positioning may therefore convert focal CAR-T engagement into a more broadly distributed intratumoral response.

Chemokine mismatch can produce a spatial failure even when antigen recognition remains intact: while CAR-T cells may eliminate accessible antigen-positive targets, tumor core regions may receive insufficient infiltration of effector cells for durable control (92). CAR-T-cell accumulation may therefore remain spatially restricted rather than extending throughout the entire lesion (92).

Persistent FAS-FASL signaling can additionally restrict T-cell persistence in selected settings (93–96). Effective tumor elimination therefore requires both antigen-specific cytotoxicity and a chemokine environment that supports distributed recruitment and positioning.

### 4.2 Stromal and vascular exclusion

A dense extracellular matrix imposes a physical constraint independent of receptor-ligand matching. CAR-T cells migrate inefficiently through collagen-rich tissue, leaving hypoxic tumor core regions particularly difficult to reach. Their distribution may remain concentrated near blood vessels or at the tumor margin rather than dispersed throughout the lesion (4–6, 20).

Cancer-associated fibroblasts deposit collagen and other extracellular matrix proteins and create restrictive physical and biochemical barriers to T-cell migration (4–6, 20). CAF-derived CXCL12 can spatially exclude CXCR4^+^ T-cells from tumor cell nests; at high local concentrations, CXCL12 may also induce immune repulsion rather than productive migratory behavior (20, 64, 65). CXCL12-CXCR4 signaling can additionally support tumor cell survival and treatment resistance, although the downstream mechanism varies by tumor type (97, 98). Furthermore, CXCL12 can also promote MDSC accumulation, further limiting T-cell access and function (99).

Vascular entry is a distinct obstacle for CAR-T cells and other cytotoxic lymphocytes. Hypoxia may impair CAR-T-cell activity (11), while tumor-associated endothelium often combines poor chemokine presentation with reduced expression of the endothelial adhesion molecules ICAM1 and VCAM1, limiting T-cell extravasation (100–102). VEGF-driven vascular dysfunction further restricts immune cell trafficking (103).

Local suppression may also originate from the tumor endothelium. Endothelial expression of PD-L1 or FasL, together with selective recruitment of regulatory or myeloid cells, can contribute to this effect (104, 105). Thus, vascular dysfunction represents both physical and immunological barriers to effective distribution of CAR-T cells within solid tumors.

## 5 Chemokines at the interface of CAR-T cell killing and regulated tumor cell death

Antigen recognition at the immune synapse elicits perforin and granzyme release, activation of death receptor pathways, and secretion of IFNγ and TNF by the CAR-T cell (106). Beyond directly inducing tumor cell death, IFNγ and TNF reshape the tumor microenvironment by altering chemokine output from malignant and stromal cells (18, 22). These changes influence the repertoire of immune cell populations and their spatial distribution (22). Chemokines can therefore organize the antitumor response without directly executing tumor cell death. Local CAR-T-cell activation and tumor cell killing may amplify local immunity by recruiting and activating effectors beyond those initially engaged by CAR-T cells. Conversely, incomplete cell death may sustain cytokine and chemokine production, promoting chronic inflammation, immune suppression, and therapeutic resistance (1, 18, 32, 33, 107).

Evidence from other treatments illustrates how tumor cell stress and immunogenic cell death can remodel local inflammatory signals. In bladder cancer models, mitomycin C induced immunogenic cell death and promoted dendritic-cell maturation through inflammasome-dependent IL-1β secretion (108). Ionizing radiation induced CXCL16 in human breast cancer cells and several murine tumor models, with CXCL16-dependent effector T-cell recruitment demonstrated in irradiated mouse breast tumors (109). Tissue damage can also modify chemotactic cues: fully reduced HMGB1 forms a heterocomplex with CXCL12 that signals through CXCR4 and recruits inflammatory mononuclear cells (110). Although this mechanism has not been demonstrated during CAR-T cell therapy, it illustrates how tumor damage can remodel the chemokine landscape encountered by infiltrating cells.

### 5.1 CAR-T cell-derived TNF and IFNγ signaling

Following CAR engagement, T-cell-derived IFNγ and TNF can act not only on the antigen-bearing target but also on neighboring tumor and stromal cells. In susceptible tumor cells, CD4^+^ CAR-T-cell-derived IFNγ can contribute to bystander killing, whereas in surviving cells cytokine exposure can induce inflammatory transcriptional programs, including chemokine production (18, 24–28, 111). Binding of IFNγ to IFNγR activates the JAK-STAT1-IRF1 axis, promoting antigen processing and presentation and inducing CXCL9, CXCL10, and CXCL11. Through CXCR3, these chemokines recruit effector T-cells and NK-cells (18, 19, 34, 41). However, TNFR1 signaling is context dependent: TNF can activate NF-kB-dependent inflammatory and survival programs or promote assembly of FADD-caspase-8-containing death complexes. Thus, TNF exposure can lead to survival signaling or death-complex formation according to the tumor cell’s pre-existing signaling state and death-pathway competence (24–26).

CAR-T-cell studies support distinct roles for these cytokines. IAP antagonism with birinapant sensitized tumor cells to CAR-T-cell-derived TNF and enhanced antitumor activity (112). Loss of IFNγR signaling impaired CAR-T-cell killing and weakened adhesion-associated interactions in solid, but not hematological, tumor models (113). In an EGFR-directed CAR-T-cell model, IFNγ-induced ICAM-1 expression enabled a second phase of CAR-T-cell infiltration into tumor islets (114). Together, these findings link cytokine responsiveness to both tumor cell killing and tissue access.

The two cytokine pathways also interact with each other. IFNγ can change the transcriptional state of a tumor cell and may lower its threshold for TNF-mediated killing while increasing chemokine production. In infection and cytokine models, combined TNF and IFNγ activated STAT1-IRF1 and FADD-caspase-8 signaling and produced a mixed inflammatory death response (27). This provides a mechanistic precedent for cytokine cooperation in cell death signaling. In a death-competent cancer cell, convergent IFNγ and TNF signaling may reinforce cytotoxicity; in a resistant cell, however, it may sustain an inflammatory transcriptional program without eliminating the target (27, 31, 33, 107).

### 5.2 Tumor cell death pathway competence

In tumor cells, death receptor signaling can initiate extrinsic apoptosis when FADD-caspase-8 signaling is intact (24, 29). Reduced caspase-8 activity can permit RIPK1-RIPK3-MLKL-dependent necroptosis, provided that the tumor cells retain functional RIPK3 kinase and the executioner pseudokinase MLKL (31, 33). Pyroptosis requires expression and proteolytic activation of an appropriate gasdermin (31, 33). PANoptosis is mediated by multiprotein PANoptosomes that coordinate caspase- and receptor-interacting protein kinase-dependent signaling. It should not be used as a collective term for coincident activation of apoptotic, necroptotic, and pyroptotic pathways, and it has not been demonstrated in the CAR-T-cell studies discussed here (30). Apoptosis, necroptosis, and pyroptosis therefore represent distinct potential fates of CAR-T-cell-targeted tumor cells.

CAR-T cells themselves can undergo FAS-dependent loss, which may limit their persistence (95, 96). The same cytokine environment may therefore produce apoptosis in one tumor, lytic death in another, and persistent inflammatory signaling with little cell loss in a third.

In B-cell malignancy models, Both et al. identified a necroptotic component of tumor cell killing mediated by CAR-T cells and T-cell-engaging bispecific antibodies, accompanied by the release of immunomodulatory factors. In the tested assays, this killing was independent of TNF and IFNγ (115). Separately, Liu et al. showed that granzyme B secreted by CAR-T cells activated caspase-3 in tumor cells, which subsequently triggered GSDME-dependent pyroptosis (116). Together, these studies demonstrate context-specific mechanisms of tumor cell death rather than CAR-T-cell-induced PANoptosis.

The competing NF-kB survival program is equally relevant. TNFR1-dependent NF-kB activation can induce c-FLIP, BCL-XL, cIAPs, and inflammatory mediators, thereby preserving a transcriptional response to TNF while preventing efficient death execution (24–26). Such a tumor may continue to produce chemokines despite surviving immune attack. This uncoupling is important for CAR-T therapy: chemokine production alone does not indicate productive tumor killing, and death-resistant cancer cells may continue to sustain local inflammatory signaling despite failed clearance (1, 33, 107).

### 5.3 Immunogenic cell death and chemokine-dependent amplification

The morphology of dying tumor cells alone cannot establish whether the resulting cell death is immunogenic. Plasma-membrane integrity is often preserved during apoptosis until the late stages, whereas necroptosis and pyroptosis are lytic modes of cell death. Immunogenicity depends on the release or exposure of tumor-derived antigens together with immune-stimulatory signals. Cellular stress or death can expose or release ATP, HMGB1, and nucleic acids, which may be recognized by innate immune sensors and promote activation of antigen-presenting cells (18, 32, 33). In this context, DAMPs primarily provide activation and maturation cues, whereas chemokine gradients regulate immune-cell recruitment and local retention.

When DAMP-mediated activation coincides spatially with chemokine-directed recruitment and retention, dendritic cells can acquire tumor-derived material, cross-present tumor antigens, and prime or restimulate endogenous CD8^+^ T-cells (18, 32, 33). CXCL9, CXCL10, and CXCL11 also draw additional CXCR3^+^ lymphocytes into the same region. Macrophages and nearby CAR-T cells may accumulate in this local niche. Tumor cell death can therefore provide both antigen and inflammatory signals, allowing the response to extend beyond the first antigen-bearing target encountered by a CAR-T cell (18, 32–35). This extension is not guaranteed: tumors that remain responsive to IFNγ and TNF but cannot execute death may continue to produce chemokines without comparable antigen release or tumor cell clearance.

Direct CAR-T-cell studies illustrate both the potential benefits and risks of inflammatory remodeling. In a lung adenocarcinoma model, oxaliplatin/cyclophosphamide conditioning induced macrophage gene expression of T-cell-recruiting chemokines, increased CAR-T-cell infiltration, and improved antitumor efficacy when combined with checkpoint blockade (117). Conversely, CAR-T-cell-induced, GSDME-dependent tumor pyroptosis activated macrophages and contributed to cytokine release syndrome (116). Thus, inflammatory tumor cell death does not necessarily translate into beneficial chemokine-mediated amplification of the antitumor response.

**Figure 2** summarizes this proposed sequence of events. CAR engagement triggers local IFNγ and TNF release. One arm of the circuit is the induction of CXCL9, CXCL10, and CXCL11 in tumor cells exposed to these mediators. The fate of the target then reflects which death machinery remains intact, with apoptosis, necroptosis, or pyroptosis as possible outcomes. When dying cells release DAMPs, innate responses can be engaged in adjacent tissue. At the same time, chemokine gradients draw dendritic cells, macrophages, endogenous CD8^+^ T-cells, and other CAR-T cells into the area. The individual steps are supported by studies of tumor immunity and inflammatory cell death, but they have not been demonstrated as one continuous circuit in a CAR-T-treated solid tumor model. Therapeutically, efficient tumor cell death should be coupled to a chemokine program that supports antigen presentation and sustained effector cell recruitment; simply increasing inflammation is not enough. Without this coupling, inflammation may persist despite incomplete tumor clearance, favoring chronic CAR-T-cell stimulation and possible premature exhaustion discussed in the next section.

**Figure 2:**
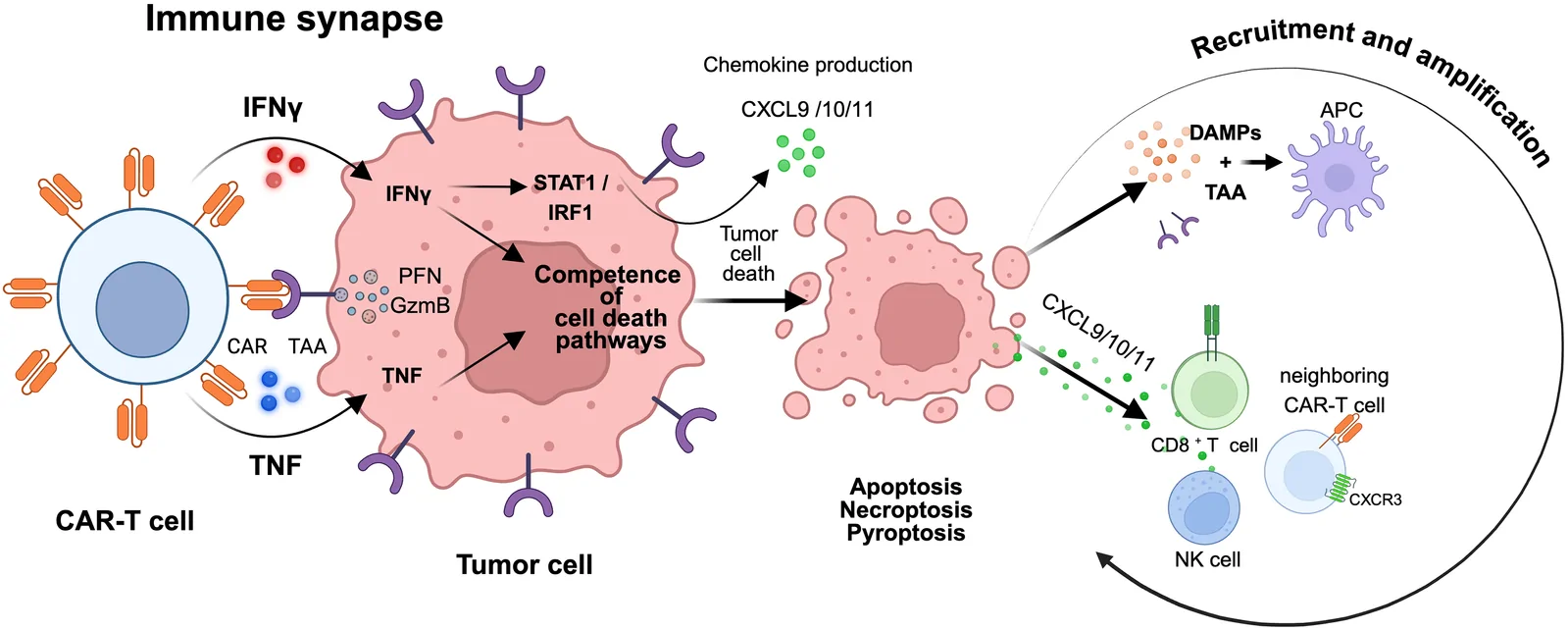
Proposed coupling of CAR-T cell activation, tumor cell death competence, and local immune amplification. CAR engagement delivers perforin and granzyme B and releases IFNγ and TNF. IFNγ-responsive STAT1/IRF1 signaling can induce CXCL9, CXCL10, and CXCL11, whereas the outcome of synaptic and cytokine-mediated stress depends on the tumor’s available death machinery and opposing survival programs. When death is executed, tumor antigens and DAMPs can support antigen-presenting cell activation, while CXCL9/CXCL10/CXCL11 recruit CXCR3-positive effectors. These parallel processes may amplify a local response. Apoptosis, necroptosis, and pyroptosis are alternative, context-dependent outcomes. APC, antigen-presenting cell; DAMP, damage-associated molecular pattern. GzmB, granzyme B; PFN, perforin; TAA, tumor-associated antigen.

## 6 Chemokines, persistence, and CAR-T cell dysfunction

Chemokine gene expression varies with CAR design and T-cell functional state. Under chronic stimulation, Selli et al. identified distinct dysfunctional populations among CD28- and 4-1BB-based CAR-T cells, characterized by CCL3 and CCL5 gene expression, respectively (118). In a study of endogenous T-cells in non-small-cell lung cancer, Thommen et al. identified PD-1^high^ CD8^+^ T cells that produced CXCL13 despite reduced conventional effector cytokine production. In this context, CXCL13 was associated with recruitment of immune cells to tertiary lymphoid structures (119).

CAR-T cells require inflammatory signaling to function, yet prolonged antigen exposure remains a major driver of exhaustion. Continued TNF and IFNγ signaling may also contribute to this loss of function. The resulting phenotype commonly includes poor IL-2-dependent proliferation, lower cytokine output, and less efficient target-cell killing. Gene expression of PD-1, TIM-3, LAG-3, and TIGIT also increases, placing further inhibitory pressure on cytotoxic activity (120, 121). Repeated rounds of antigen-driven proliferation may shorten telomeres, cause DNA damage, and contribute to terminally differentiated or senescence-like states (122, 123). In this context, chemokine networks are increasingly viewed as part of these dysfunctional adaptation programs rather than as passive regulators of leukocyte transport (124).

CAR-T-cell-intrinsic fitness can amplify the effects of an immunosuppressive microenvironment. T-cells from previously chemotherapy-treated patients may contain fewer naïve cells and yield CAR-T cells with reduced cumulative killing capacity upon repeated antigen stimulation (125). Chemotherapy can also leave durable mutational signatures and alter the clonal structure of normal blood cells (126). Chronic antigen exposure can promote exhaustion, whereas metabolic and proliferative stress may induce senescence-like states. These processes both impair CAR-T function but are biologically distinct (127–131).

Intracellular NF-kB signaling plays a central role in these adaptation programs. TNF-driven IKK-NF-kB signaling and IFNγ-driven JAK-STAT1 signaling may initially support increased inflammation and immune cell recruitment. NIK (NF-kB-inducing kinase) has a metabolic role in T-cells in addition to its established place in non-canonical NF-kB signaling. This metabolic effect does not require transcriptional NF-kB activity: NIK stabilizes hexokinase 2 (HK2) through ROS/G6PD-NADPH-dependent control of autophagic HK2 degradation (132). Such support may help cytotoxic T-cells function during acute inflammation, while inflammatory signaling promotes tumor cell death. In tumor cells, however, persistent NF-kB activity can induce c-FLIP, BCL-XL, and cIAPs, as well as CCL2 and CXCL8 (24–26). Recruitment of monocytes, TAMs, and MDSCs through CCL2, and of neutrophils and MDSCs through CXCL8, reinforces suppressive niches that impair CAR-T cell persistence and function (51, 56), as summarized in **Figure 3**. Prolonged exposure to IFNγ can further promote compensatory resistance programs involving STAT1, IRF1, PD-L1, and IDO1 (28). In various models of melanoma and other solid tumors, prolonged IFNγ signaling has been associated with stable epigenetic remodeling and the acquisition of immune-resistant transcriptional states (133, 134).

**Figure 3:**
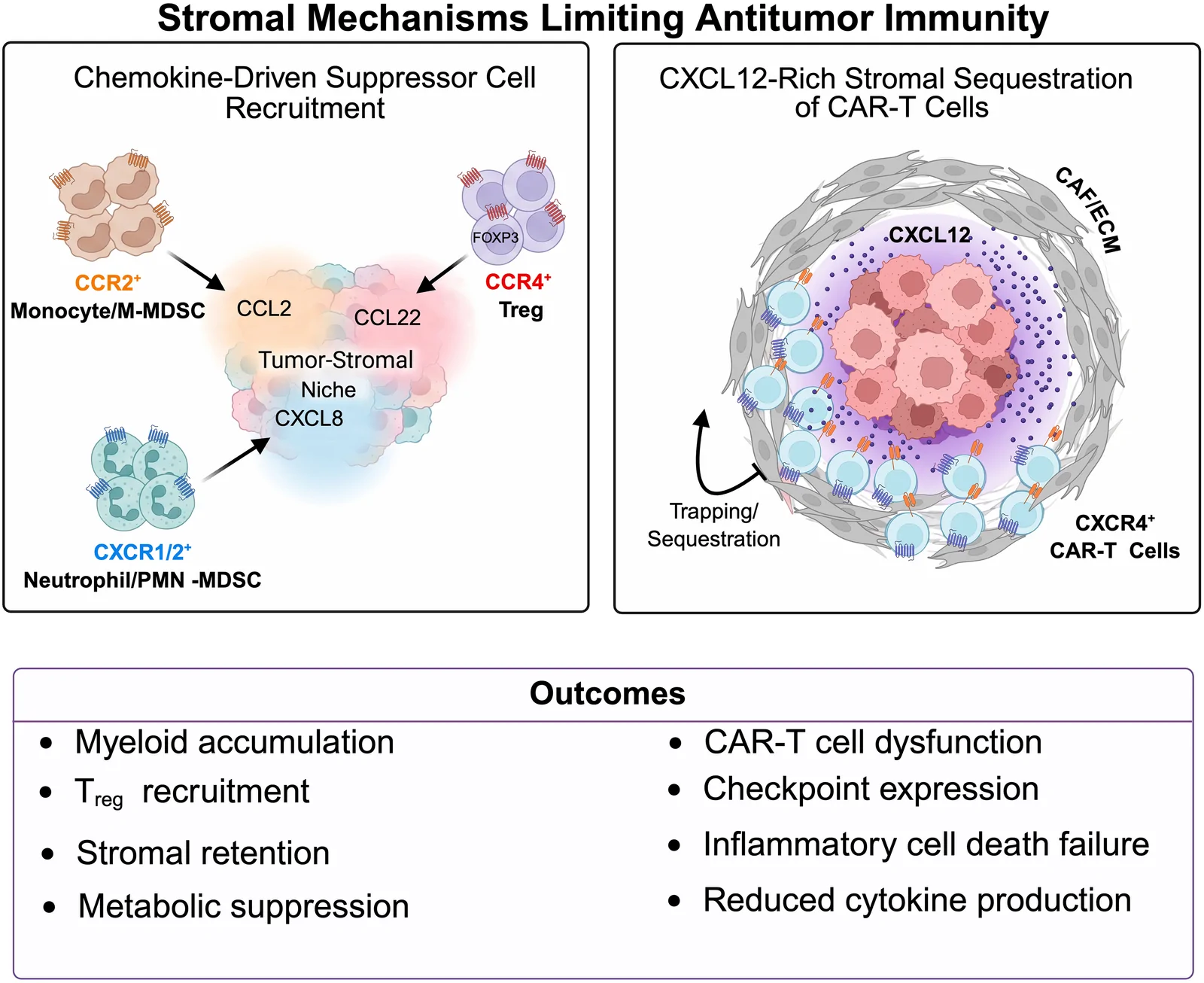
Suppressive chemokine circuits in a resistant tumor niche. CCL2 recruits CCR2-positive monocytes and monocytic MDSCs, CCL22 recruits CCR4-positive regulatory T-cells, and CXCL8 recruits CXCR1/2-positive neutrophils and polymorphonuclear MDSCs. Recruited monocytes may subsequently differentiate into TAMs. In parallel, CAF-associated CXCL12 can retain CXCR4-positive CAR-T cells in an ECM-rich stromal compartment. Together, these pathways can increase suppressive cell accumulation and reduce CAR-T access and function. Ligand sources and biological effects are highly context-dependent. CAF, cancer-associated fibroblast; ECM, extracellular matrix; MDSC, myeloid-derived suppressor cell; TAM, tumor-associated macrophage; Treg, regulatory T cell. M-MDSC, monocytic myeloid-derived suppressor cell; PMN-MDSC, polymorphonuclear myeloid-derived suppressor cell.

This persistent stimulation can also negatively affect CAR-T cells by triggering transcriptional and epigenetic exhaustion programs. Activation of transcription factors including TOX and NR4A family members promotes chromatin remodeling leading to a dysfunctional state of CAR-T cells and their declining effector capacity (135). Inflammatory chemokines may maintain these dysfunctional interactions by persistently recruiting CAR-T cells to tumor-associated suppressive niches enriched in hypoxia, inhibitory cytokines, and myeloid-derived inflammatory mediators (2).

Single-cell studies indicate that CAR-T-cell products comprise heterogeneous memory, effector, activated, and exhaustion-associated states rather than a simple functional-versus-dysfunctional dichotomy. In an APRIL-based CAR-T-cell product from three healthy donors, only a small fraction of antigen-responsive CAR-expressing cells acquired an exhaustion-associated transcriptional profile after acute stimulation, highlighting marked intraproduct heterogeneity (136). In that study, cells with high exhaustion scores also expressed *CCL1, CCL3, CCL4*, and *CCL5* (136).

In a phase II study of CD19-directed CAR-T cells, effector-like, polyfunctional CD8^+^ CAR-T cells with low dysfunctional signatures were associated with clinical response, whereas prolonged *ex vivo* expansion was associated with a lower proportion of this subset (137). Conversely, responder-associated effector CAR-T cells expressed *CCL1, CCL3, CCL4*, and *XCL1* (137). Another single-cell study linked early-memory and type-2 functional features, rather than activation or coinhibitory marker expression alone, to durable remission (138). Chemokine expression is therefore not specific to either functional or dysfunctional states and should be interpreted alongside cytotoxic, memory, proliferative, and inhibitory receptor programs.

In this respect, inflammatory but death-resistant tumors may be particularly important drivers of CAR-T cell dysfunction. Tumors with intact inflammatory signaling pathways but impaired apoptosis or necroptosis may remain responsive to TNF and IFNγ cytokine signaling without undergoing effective clearance. Under these conditions, chronic cytokine and chemokine production would prolong the interaction with CAR-T cells while simultaneously enhancing suppressive inflammatory programs. A mechanistic precedent in acute lymphoblastic leukemia (ALL) showed that impaired tumor cell death signaling can contribute not only to immune resistance but also to progressive CAR-T cell dysfunction despite retained target antigen expression (29), as conceptually illustrated in **Figure 4**.

**Figure 4:**
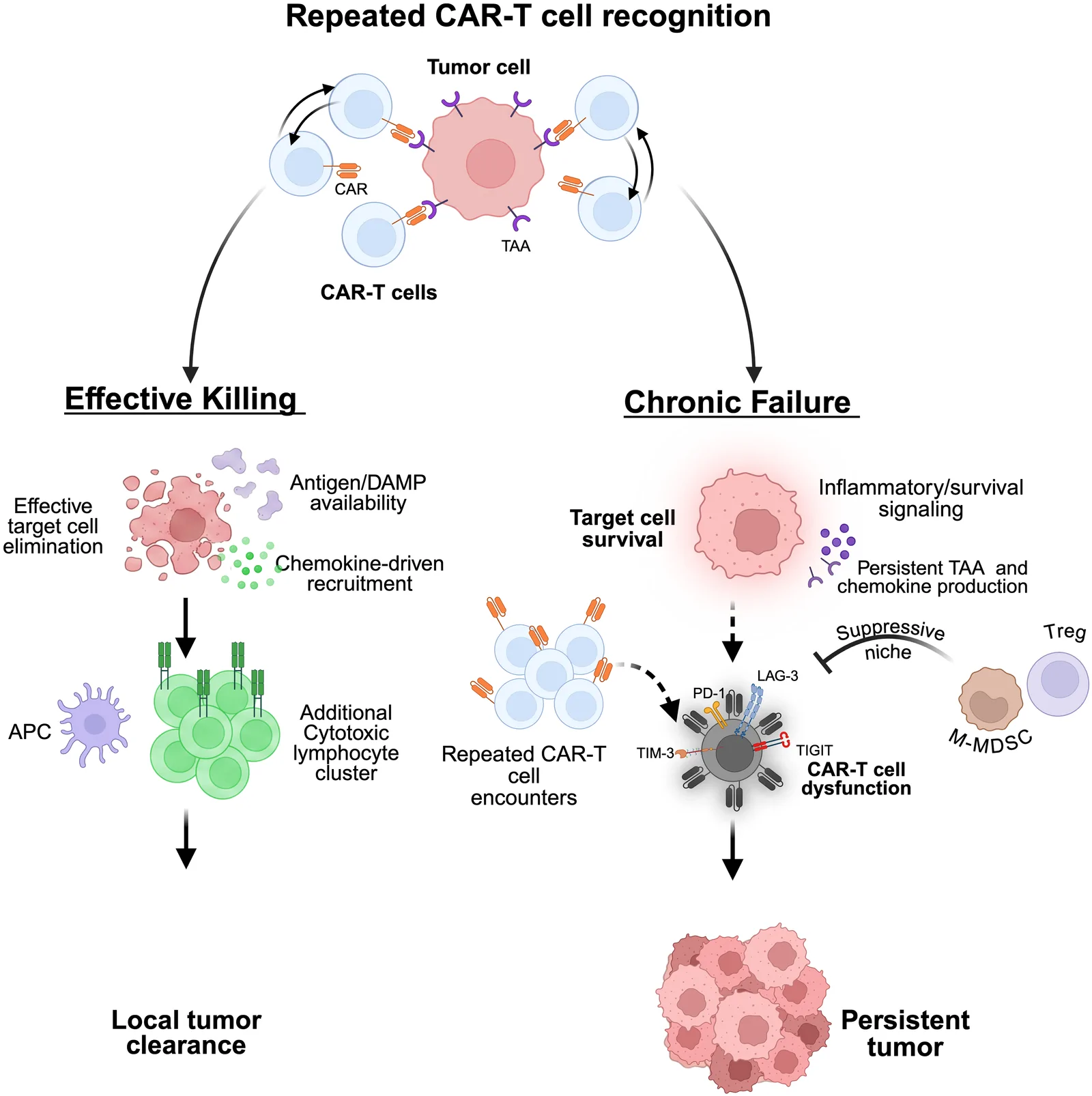
Tumor cell death competence separates productive clearance from chronic inflammatory resistance. When the target executes death efficiently, antigen/DAMP availability and effector-recruiting chemokines can support antigen-presenting-cell activation, additional effector recruitment, and local clearance. When the tumor survives despite cytokine responsiveness, persistent antigen and chemokine production can sustain repeated CAR-T encounters and a suppressive niche. The co-inhibitory receptors shown are illustrative; their gene expression alone does not establish CAR-T dysfunction, which requires functional assessment. The chronic CAR-T-specific feedback shown is conceptual. DAMP, damage-associated molecular pattern. APC, antigen-presenting cell; M-MDSC, monocytic myeloid-derived suppressor cell; TAA, tumor-associated antigen; Treg, regulatory T cell.

Durable CAR-T cell activity also depends on the formation or maintenance of memory-like T-cell populations. Persistent antigen stimulation and exhaustion can compromise this process. Approaches that preserve memory formation while maintaining antitumor activity are therefore an important design goal, as discussed below (120, 135).

## 7 Chemokine pathway engineering strategies for enhanced CAR-T cell therapy

Improved trafficking should be pursued alongside preservation of memory-like CAR-T-cell states. Manufacturing and circuit design can limit prolonged *ex vivo* expansion and repeated stimulation, while locally inducible rather than constitutive chemokine or cytokine release may reduce chronic signaling (2, 120, 135). Single-cell analyses of clinical CAR-T-cell products further associate early-memory states and IL-4/IL-13-associated type-2 functional programs with durable responses (138, 139). Thus, an effective trafficking module should improve tumor access without compromising the proliferative reserve required for CAR-T-cell persistence and repeated challenge.

### 7.1 Engineering chemokines and matched trafficking programs

Chemokine receptor matching has been reviewed extensively (20); representative examples are therefore summarized briefly here. CCR2b expression improved migration of mesothelin-directed CAR-T cells toward CCL2-producing tumors and enhanced xenograft control (89). CXCR1 or CXCR2 expression similarly improved trafficking and activity in CXCL8-rich glioblastoma, pancreatic-cancer, and ovarian-cancer models (90), and CXCR2 was also combined with an αvβ6-directed CAR (140). These benefits are context dependent. CCR5 can support recruitment, but CCL5-CCR5 signaling may also promote angiogenesis or suppressive myeloid-cell accumulation; consequently, receptor addition, ligand deletion, or pharmacological blockade can produce different outcomes according to the cellular source of CCL5 (141–144).

An alternative strategy is to engineer the chemokine itself. Several tumor microenvironments express high levels of the DPP-4 sequence-specific protease that removes the two N-terminal residues of CXCL9 and CXCL10. DPP4-generated truncated forms retain CXCR3 binding but lose agonist activity. Addition of an N-terminal glutamine produced DPP-4-resistant CXCL9-Fc and CXCL10-Fc agonists that enhanced IFNγ-high effector responses and improved tumor control in preclinical models (145).

Separately, an N20K substitution in CXCL10 increased glycosaminoglycan binding and enhanced T-cell migration in trans-endothelial and Boyden-chamber assays (146). Together, these studies show that chemokine stability and matrix binding can be engineered to support chemotactic activity without altering the chemokine receptor repertoire of the infused T-cells.

### 7.2 Chemokine- or cytokine-armored CAR-T cells can improve persistence

It has been shown that CAR-T cells co-expressing IL-7 and CCL19 recruited endogenous T-cells and dendritic cells and improved survival of CAR-T cells and their accumulation in preclinical tumors (147, 148). The IL-7/CCL21 strategy should also be considered because both CCL19 and CCL21 signal through CCR7. In a direct preclinical comparison of IL-7/CCL21- and IL-7/CCL19-expressing CAR-T cells, the former produced superior tumor control compared to IL-7/CCL19 CAR-T cells in three solid tumor models. Importantly, this result was achieved under non-lymphodepleting conditions. This difference was notable, as the two chemokines showed similar chemotactic activity *in vitro* whereas tumor control did not differ significantly in the lymphodepleted pancreatic-tumor model (20, 149).

IL-7/CCL3-expressing CAR-T cells represent another preclinical approach as they were shown to promote tissue-resident-memory-like phenotypes and persistence across several preclinical tumor models (150).

### 7.3 Combination therapies that remodel the tumor chemokine landscape

Combination therapy can also alter the chemokine landscape. Oncolytic viruses can convert poorly inflamed tumors into lesions with stronger inflammatory signaling and chemokine production. cGAS senses cytosolic DNA and activates STING through cGAMP, while related innate sensors respond to nucleic acids derived from viruses or tumor cells, initiating type I interferon programs in the surrounding tissue (151, 152). Lysis of infected cancer cells adds DAMPs that may support dendritic cell activation, antigen cross-presentation, and effector cell recruitment. For example, an engineered CDK4/6 inhibitor-armed oncolytic adenovirus triggered tumor cells to release CCL5 and recruit CD8^+^ T-cells. It also reduced T-cell exhaustion associated with repeated viral treatment and enhanced CAR-T cell activity (153). An oncolytic adenovirus expressing *CCL19* additionally promoted CCR7-dependent CAR-T cell migration and tumor control in an ovarian cancer xenograft (154). This strategy does not require the CAR-T cell product itself to secrete the chemokine. In principle, a replicating virus can provide a local and renewable chemokine source and may reduce dependence on the number of pioneer CAR-T cells that initially reach the tumor.

Notably, further studies have shown that increasing intratumoral chemokine abundance on its own is not uniformly sufficient to improve T-cell trafficking. In one study, an oncolytic vesicular stomatitis virus engineered to express CXCL9 generated a steep tumor-to-blood gradient but did not measurably increase trafficking of transferred or endogenous T-cells in several tumor models (155). In another study, vaccinia-virus-mediated delivery of CXCL11 increased intratumoral T-cell recruitment and antitumor activity, whereas constitutive CXCL11 secretion by CAR-T cells failed to increase subsequent recruitment of CAR-T cells and instead reduced CAR-T-cell function (156). Collectively, these contrasting results point to the importance of chemokine source, timing, concentration, and duration as design variables rather than assuming that greater ligand abundance is invariably beneficial.

Ionizing radiation can also strengthen local recruitment signals by increasing CXCL16 production in tumor cells (109). CXCR4 blockade and CAF targeting can disrupt CXCL12-rich stromal sequestration and reduce T-cell exclusion (65). In a separate lymphoma model, gene co-expression of CCR4 with a CD30-directed CAR enhanced antitumor activity (157). CXCR4 blockade combined with checkpoint-receptor inhibition may also improve dendritic-cell antigen presentation and cytotoxic-lymphocyte recruitment, as reported in preclinical hepatocellular carcinoma models (158).

Together, chemokine/chemokine receptor engineering, armored chemokine/cytokine programs, and microenvironment-targeted combination strategies indicate that effective CAR-T-cell therapy in solid tumors requires not only antigen recognition but also appropriate spatial targeting, persistence, and inflammatory remodeling. Rational engineering may therefore help convert a spatially restricted immune response into coordinated tumor cell elimination across the tissue, as summarized in **Figure 5**.

**Figure 5:**
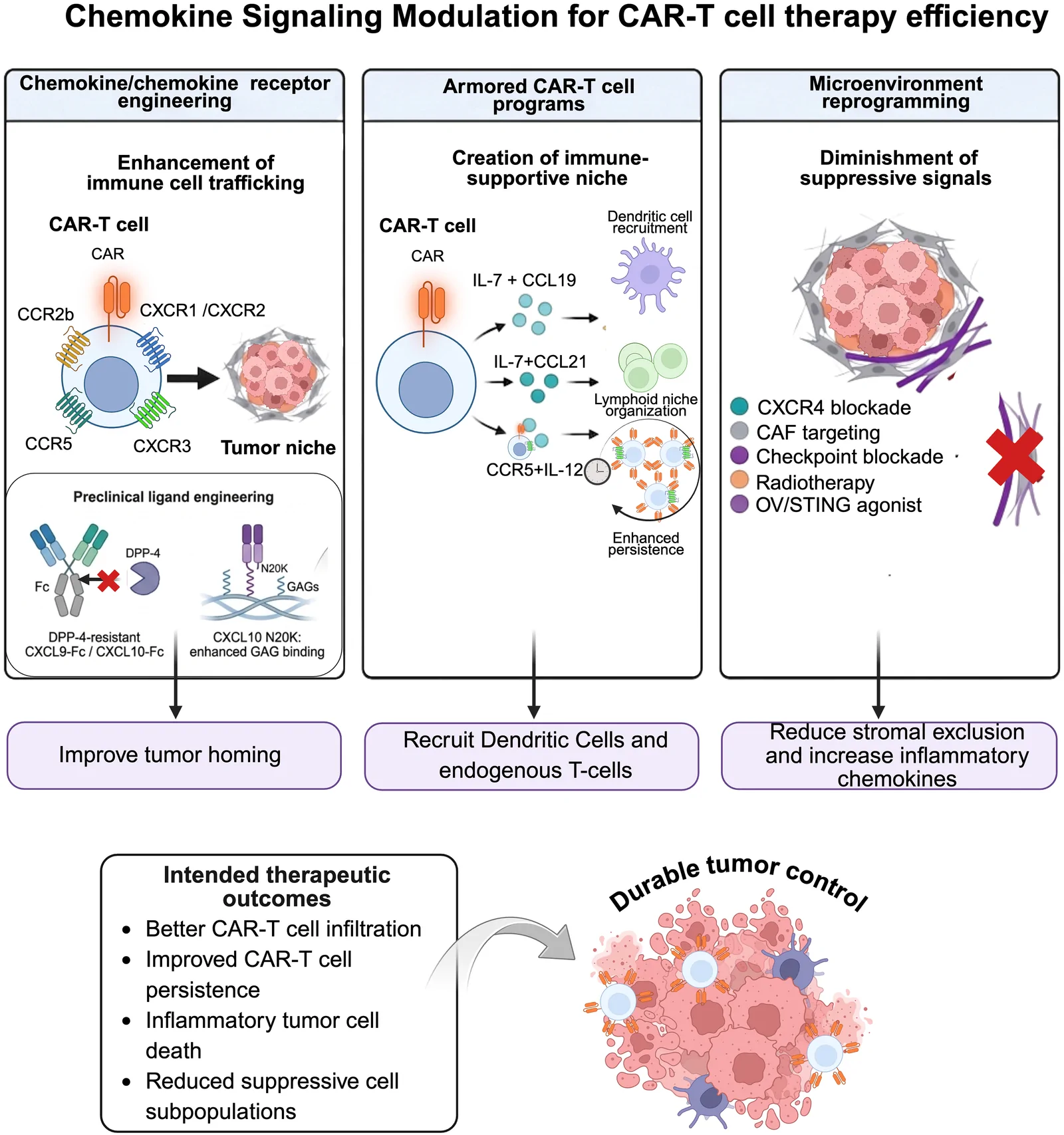
Chemokine-based strategies for improving CAR-T cell therapy in solid tumors. The left panel depicts chemokine/chemokine receptor engineering. Engineering with CCR2b, CXCR1/CXCR2, CXCR3, or CCR5 is intended to align CAR-T cell migration with chemokines produced in the lesion; these are alternative constructs, not one product expressing all four receptors. Ligand engineering includes DPP-4-resistant CXCL9-Fc/CXCL10-Fc and GAG-binding-enhanced CXCL10 variants. Because CCL5-CCR5 signaling is context dependent, it can also support angiogenesis or myeloid recruitment. The middle panel depicts separate armored products built around IL-7 plus CCL19, IL-7 plus CCL21, or CCR5 plus IL-12. The CCL21 branch represents co-expression with IL-7. In the CCR5/IL-12 co-engineered construct, CCR5 is a cell-surface receptor and IL-12 is secreted. Their purpose is to recruit dendritic cells and endogenous T-cells, promote lymphoid-like organization, or sustain the CAR-T cell population. The right panel covers microenvironment-directed interventions: CXCR4 inhibition, CAF targeting, checkpoint blockade, radiotherapy, oncolytic viruses, and STING agonists. Depending on the approach, these investigational interventions may relieve stromal exclusion, reinforce local inflammatory chemokine signaling, or do both. Intended outcomes are improved entry and persistence, more effective tumor cell killing where death competence is preserved, fewer suppressive cells, and improved tumor control. Most strategies shown are preclinical or investigational. CAFs, cancer-associated fibroblasts; STING, stimulator of interferon genes. OV, oncolytic virus.

## 8 Discussion and translational perspectives

Chemokine engineering has opened several routes for improving CAR-T cell therapy, but its impact in solid tumors is still constrained by the organization of the underlying network. Shared receptor usage creates a potential source of redundancy because several chemokines can signal through the same receptor. Blocking one ligand-receptor pair does not necessarily stop recruitment, because other chemokine routes may remain active. Even then, the change in immune cell traffic may be modest or short-lived, with little effect on tumor control overall.

A chemokine profile is dynamic rather than a stable property. It changes with tumor progression and treatment. Local inflammation, hypoxia, and immunosuppression add a spatial layer to that variation. The functional impact of a chemokine gradient may also change over time. A signal that initially recruits CAR-T cells into the tumor may subsequently sustain inflammation, attract suppressive myeloid populations, or contribute to T-cell dysfunction. Moreover, inflamed regions can coexist with immune-excluded, hypoxic, and stroma-rich niches within the same tumor. Access to a permissive niche therefore does not necessarily translate into effective control of the tumor as a whole.

### 8.1 Clinical translation: early evidence and lessons from negative studies

Clinical translation of chemokine-engineered CAR-T cells remains at an early stage. The chemokine-armed and chemokine receptor-engineered CAR-T cell products discussed here remain investigational, and the cited studies do not isolate the clinical contribution of a chemokine module from the CAR, lymphodepletion, route of administration, or other co-expressed payloads. Notably, in 39 patients with relapsed or refractory large B-cell lymphoma treated with antigen-inducible IL-7/CCL19 CD19 CAR-T cells (NCT03258047), the 3-month overall (ORR) and complete (CRR) response rates were 79.5% and 56.4%, respectively. Median progression-free survival (PFS) was 13 months, and grade 3 cytokine release syndrome (CRS) and grade 3 or higher neurotoxicity (ICANS) occurred in 12.8% and 10.3%, respectively (159). These findings establish feasibility; however, the single-arm hematological study does not demonstrate superiority over a matched conventional CAR-T-cell product or establish efficacy in solid tumors.

Early solid-tumor experience illustrates both potential activity and substantial limitations. In a heterogeneous six-patient phase I cohort treated with GPC3- or mesothelin-directed IL-7/CCL19-secreting CAR-T cells (NCT03198546), investigators reported one complete response, one partial response, and two cases of stable disease (160). However, heterogeneity in tumor type, CAR target, administration route, and dosing schedule, together with the absence of a matched control group, precludes attributing these responses specifically to IL-7/CCL19. In NCT03778346, only the first two patients treated with BCMA-directed IL-7/CCL19 CAR-T cells were reported, achieving one complete response and one very good partial response (161). These observations should therefore be regarded as preliminary feasibility signals rather than estimates of efficacy.

Chemokine receptor engineering has generated preliminary clinical evidence consistent with enhanced trafficking, but this has not consistently translated into durable disease control. In phase I study NCT03602157, conference reports described responses in 22 of 24 patients with relapsed or refractory Hodgkin lymphoma treated with CCR4-expressing CD30 CAR-T cells, with conventional CD30 CAR-T cells co-infused at selected dose levels. The study reported 16 complete responses, although median progression-free survival was 6.4 months (162). In a six-patient cutaneous T-cell lymphoma cohort, the week-6 objective response rate was 50%, no complete responses were observed, all patients subsequently required additional therapy, and the median time to next systemic treatment or death was 7.4 months (163). These findings suggest that chemokine receptor engineering alone may be insufficient to overcome antigen heterogeneity, limited persistence, or local immunosuppression. Importantly, the current evidence reflects heterogeneous and sometimes transient activity and controlled studies have not yet established the incremental benefit of the chemokine receptor module.

Clinical translation is also constrained by manufacturing complexity and assay requirements. A chemokine receptor selected on the basis of one biopsy may encounter a different ligand landscape in another lesion or after treatment. Furthermore, high ligand abundance may be nondirectional and induce receptor internalization or desensitization, or recruit suppressive myeloid populations. Additional receptor and payload cassettes must maintain stable gene expression, preserve chemotactic function after cryopreservation, and meet product-specific requirements for controlled secretion and release testing. In the 2024 Hodgkin lymphoma report, the median interval from cell collection to lymphodepletion was 58.5 days; across the full Hodgkin and cutaneous T-cell lymphoma trial cohort, seven patients with manufactured products were not infused, including three who died during the manufacturing period (162). Future trials should incorporate biomarker-based patient selection, an otherwise matched CAR-T-cell comparator, prespecified trafficking and persistence endpoints, paired tumor biopsies, and potency assays that assess both antigen-dependent cytotoxicity and ligand-directed migration.

### 8.2 Single-cell transcriptomics and spatial profiling for CAR target selection and monitoring of CAR-T cells

Single-cell RNA sequencing (scRNA-seq) enables high-resolution profiling of cellular heterogeneity and molecular states. Although flow cytometry remains a standard method for monitoring CAR-T cell immunophenotypes, its reliance on predefined marker panels limits discovery beyond the selected markers. scRNA-seq can identify cellular states and molecular programs associated with CAR T-cell expansion, persistence, and therapeutic efficacy (164). The growing availability of publicly accessible single-cell datasets from normal and diseased tissues further enables systematic identification of candidate antigens with preferential tumor expression for CAR T-cell targeting. For example, analysis of 28, 404 bone marrow cells from 15 patients with acute myeloid leukemia (AML), together with more than 500, 000 healthy cells from nine tissue types, identified colony-stimulating factor 1 receptor (CSF1R) and CD86 as candidate CAR-T cell targets based on malignant-cell enrichment and assessment of gene expression in healthy tissues; both were subsequently validated in preclinical models (165). Similarly, single-cell atlases comprising more than 300, 000 cells from 48 patients with follicular lymphoma, multiple myeloma, and B-cell acute lymphoblastic leukemia were compared with an atlas of over 3 million cells from 35 healthy tissues to rank 15 investigational targets by their expression profile similarity to approved targets (166). However, target expression did not significantly correlate with reported clinical toxicities in that analysis, highlighting the need for clinical validation and consideration of patient- and treatment-specific factors.

Spatial transcriptomic and imaging approaches can provide an additional layer of information by preserving the anatomical context of tumor and immune cells. For example, *in situ* profiling of more than 274, 000 cells using a 1, 000-gene CosMx panel in pretreatment lesions from 20 women with HPV16-positive vulvar high-grade squamous intraepithelial lesions identified baseline immune and stromal features associated with response to therapeutic HPV16 peptide vaccination (167).

More recently, single-cell spatial pharmacobiology (SSP) has emerged as a framework integrating *in situ* visualization of a fluorescently labeled therapeutic antibody with high-plex spatial proteomics to quantify drug distribution, target engagement, and tumor microenvironment architecture (168). A similar strategy could potentially be adapted to map the tissue biodistribution and localization of labeled CAR-T cells and their proximity to antigen positive tumor cells, providing a spatial dimension that is not captured by conventional single-cell transcriptomics.

Single-cell and spatial profiling can convert chemokine engineering from an empirical choice of receptor(s) into a testable patient-selection strategy. Before treatment, single-cell RNA-sequencing with surface protein measurements can identify malignant, stromal, endothelial, and myeloid sources of candidate ligands. In parallel, chemokine receptor expression and differentiation states should be defined in the apheresis material and the final CAR-T-cell product. Memory-like CD8^+^ CAR-T cells were enriched in patients achieving complete responses to axi-cel, whereas an exhaustion program was associated with poor early molecular response (169). Other product studies linked effector-like, polyfunctional CD8^+^ CAR-T populations with low dysfunction scores to response (137) and showed that only a fraction of CAR-expressing cells may mount a detectable transcriptional response to antigen (136). Importantly, these measurements can inform patient stratification and manufacturing release criteria rather than serving only as retrospective descriptors.

Spatial information is essential because transcript abundance alone does not define a functional chemokine gradient. Spatial transcriptomics and multiplex imaging can determine whether a ligand is positioned around perfused blood vessels, within tumor nests, at fibroblast-rich borders, or in necrotic and hypoxic regions, and whether engineered CAR-T cells cross these boundaries or remain trapped. A recent multimodal atlas of primary PDAC combined whole-transcriptome Visium profiling with targeted single-cell CosMx imaging (170). The atlas also included registered histology, collagen assessment, tumor-distance zones, and cell-neighborhood analysis to resolve tumor- and stroma-associated spatial programs. However, no CAR-T cell intervention or protein gradient measurement was included. Combining spatial maps with T-cell receptor or vector-transgene capture can distinguish expansion of the infused product from recruitment of endogenous lymphocytes and can separate entry, intratumoral retention, and egress. Longitudinal single-cell analysis of blood has resolved the clonal expansion, contraction, and phenotypic evolution of infused CAR-T cells (171), whereas paired blood and tumor profiling in IL-15-armored GPC3 CAR-T trials linked intratumoral CAR-T states to clinical response (172). The most informative design would integrate the infusion product, serial blood, and paired pretreatment and on-treatment biopsies from more than one lesion.

Chemokine mRNA does not measure secreted protein, proteolytic processing, glycosaminoglycan-bound presentation, or a directionally active gradient. Receptor mRNA does not establish surface abundance, internalization, or chemotactic competence either. Computational ligand-receptor inference should therefore be validated by spatial protein measurements, flow cytometry, quantitative chemokine assays, and functional migration tests. A clinically useful biomarker should be prespecified and analytically validated, for example as the proximity of ligand-producing cells to perfused vasculature and antigen positive tumor nests rather than bulk ligand expression alone. Serial sampling can then determine whether treatment removes the selected axis, induces an alternative suppressive circuit, or drives a dysfunctional CAR-T state.

Published single-cell and spatial analyses identified a spatial association between CXCR6^+^ T-cells and CXCL16-expressing myeloid cells in ICANS, with tissue-level spatial validation limited to one fatal case (173). Our exploratory patient-level reanalysis of four public CAR-T cohorts (174–177) found variable changes in CXCR6 transcript-positive cell fractions without a consistent early increase. These transcript-level findings do not establish trafficking or clinical benefit; code and results are stored in the associated repository at https://github.com/alexeysakhalin/Chemokines-as-Regulators-of-CAR-T-Cell.

### 8.3 Safety and controllability of engineered products

Safety mechanisms should be incorporated directly into engineered CAR-T-cell products. In the clinically evaluated CD19 7x19 construct, IL-7 and CCL19 gene expression was placed under control of NFAT-responsive elements and a minimal IL-2 promoter, coupling secretion to CAR activation rather than constitutive systemic release (159). Pharmacologically reversible ON- and OFF-switch CARs, including lenalidomide-controlled split or degradable receptors, offer temporal control but are still supported mainly by preclinical data (178). Such reversible systems may be preferable when transient suppression is sufficient, whereas irreversible suicide switches are reserved for severe or refractory toxicity and are intended to deplete the transferred-cell population.

In line with this, inducible caspase-9 (iCasp9) provides clinical proof of principle for a suicide switch in armored CAR-T-cell therapy. In iCasp9-engineered CAR-T cells, caspase-9 is fused to a rimiducid-binding dimerization domain. Once caspase-9 molecules are forced to dimerize, they activate the apoptotic cascade and rapidly eliminate the modified cells. In phase I studies of IL-15-armored GPC3 CAR-T cells, three patients with prolonged cytokine release syndrome or hypoxia received rimiducid. iCasp9 activation rapidly reduced circulating engineered cells and inflammatory cytokines and was followed by clinical improvement (172). However, suicide switches act only after activation and therefore cannot be assumed to prevent the initial tissue injury.

Microenvironment-responsive regulation provides an additional layer of safety control. Hypoxia-responsive CAR gene expression reduced activation under normoxic conditions in preclinical models (179). In a 43-patient phase I study of the hypoxia-responsive CEA-directed product PC13 (NCT05396300), the predefined safety endpoint was met; however, 20.9% of patients developed grade 3 diarrhea and 76.7% experienced grade 1–2 cytokine release syndrome. Objective response rates were 23.5% after intraperitoneal administration and 8.0% after intravenous administration (180). These findings suggest that microenvironment-responsive transcription may widen the therapeutic window but does not eliminate on-target, off-tumor toxicity or route-dependent differences in exposure. A robust safety architecture should therefore combine activation- or microenvironment-dependent gene expression with dose and route control, prospective monitoring for organ-specific inflammation, and an independently actionable OFF or suicide switch.

Circulating chemokines and cytokines may complement tissue-based monitoring (181). CXCL9 and CXCL10 are induced by IFNγ (34), whereas elevated CXCL8/IL-8 is associated with myeloid-rich inflammation and reduced benefit from immune checkpoint blockade. The utility of circulating CXCL8/IL-8 for CAR-T-cell monitoring remains uncertain (60, 61). Persistent inflammatory cytokine exposure may likewise indicate unresolved tumor-immune interactions and a microenvironment permissive to CAR-T-cell dysfunction. Serial measurements could therefore provide a minimally invasive readout of inflammatory adaptation, although they cannot resolve the spatial organization of chemokine gradients. Candidate spatial, transcriptional, and circulating biomarkers are summarized in **Table 1**.

**Table 1:** Candidate chemokine biomarkers and immune signatures for translational studies of CAR-T cell therapy in solid tumors.

Candidate marker or signature	Source or assay compartment	Biological feature captured	Possible translational interpretation	Proposed sampling time points	Key reference(s)
CXCL9/CXCL10/CXCL11	Dendritic cells, macrophages, tumor cells	CXCR3-mediated entry of CD8+ T-cells and NK-cells	Inflamed tumor feature; reported association with favorable immunotherapy response	Before therapy and early after treatment starts	(19, 34, 42)
CXCR3 signaling	Activated effector T-cells	Effector cell migration into tumor tissue	Supports infiltration; possible relevance to treatment response	Baseline receptor assessment and CAR-T product characterization	(19, 34)
CCL2-CCR2 axis	Tumor cells, TAMs, stromal cells	Monocyte recruitment; TAM/MDSC accumulation	Suppressive myeloid-rich resistant niche	Before treatment; repeat at progression or relapse	(17, 20, 51, 88)
CXCL8-CXCR1/CXCR2	Tumor cells, neutrophils, other myeloid cells	Neutrophil/MDSC influx; angiogenesis	Reduced checkpoint benefit; CAR-T relevance not established	Serum and tumor at baseline and early on treatment	(56, 57, 60, 61)
CXCL12-CXCR4	CAFs, lymphatic endothelium, stroma	Stromal exclusion, recruitment, retention, or egress	Direction depends on concentration, spatial organization, and cellular source	Spatial profiling before and during treatment	(20, 64, 65, 67)
IFNγ-response signature	Tumor and immune cells	Antigen presentation and inflammatory activation	Candidate predictive or pharmacodynamic feature; requires validation in CAR-T therapy	Baseline and early on treatment	(42)
NF-kB inflammatory-survival program	Tumor cells and TAMs	Chronic inflammation with anti-apoptotic adaptation	Potential resistance state despite chemokine production	At progression or relapse	(24–26)
Spatial CXCL9/CXCL10 niches	Dendritic cells or macrophages near CD8+ T-cells	Local inflammatory amplification	Candidate feature of productive immunity	Spatial transcriptomics or multiplex imaging before and on treatment	(18, 85)
Circulating CXCL9/CXCL10	Serum or plasma	Systemic IFNγ-driven activation	Proposed pharmacodynamic readout; requires validation in CAR-T cohorts and does not resolve spatial gradients	Early and serially	(34, 181)
Serum IL-8/CXCL8	Serum or plasma; tumor and myeloid compartments	Systemic myeloid-associated inflammation	Candidate marker of myeloid inflammation; predictive value in CAR-T therapy remains unvalidated	Baseline and early on treatment	(60, 61)
CAR-T product state	Infusion product and serial blood; single-cell assays	Memory, effector, activated, and dysfunction-associated heterogeneity	Product-quality and response-stratification candidate	Before infusion and serially after treatment	(136–138, 169)
CXCL16 proteoform and location	Tumor, endothelium, and myeloid compartments; protein and spatial assays	Membrane adhesion versus soluble chemotaxis	Bulk RNA or total protein is insufficient to infer function	Pretreatment and on-treatment tumor tissue	(44–46)

Synthetic cytokine-receptor pairs provide a complementary strategy for cell-restricted support. In a murine model, a human orthogonal IL-2/IL-2Rβ pair selectively expanded receptor-matched CAR-T cells and enhanced anti-leukemic activity (182). This establishes a proof of principle for orthogonal, cell-restricted survival or expansion signals that could in future be combined with trafficking engineering, although whether such systems reduce off-target inflammation in patients remains to be determined.

### 8.4 Limitations and future directions

The field has four central limitations. First, most chemokine-engineering data come from xenografts that do not reproduce human chemokine cross-reactivity, endogenous immune recruitment, or organ-specific inflammation. Second, clinical evidence consists mainly of small, single-arm, heterogeneous early-phase studies in which chemokine receptors or ligands are bundled with cytokines, different CAR targets, local delivery, repeated dosing, or additional genetic changes, preventing causal attribution. Third, bulk or single-time-point expression does not establish a stable, spatially directional gradient, and no validated threshold identifies patients likely to benefit from a particular receptor-ligand pair. Fourth, long-term off-tumor trafficking, inflammatory toxicity, clonal behavior, manufacturing consistency, and cost also form significant constraints.

Priority studies should combine humanized or immunocompetent models with patient-derived organotypic systems, followed by prospective biomarker-selected trials comparing the engineered product with an otherwise matched CAR-T-cell control. These trials should include multisite baseline sampling, longitudinal single-cell and spatial profiling, direct assessment of intratumoral CAR-T-cell localization, and prespecified pharmacodynamic evidence of engagement of the intended ligand-receptor axis. Standardized chemotaxis and secretion potency assays, reversible control or suicide modules, and long-term follow-up should also be incorporated into development. Non-response, transient response, manufacturing attrition, and organ-specific toxicity should be reported alongside conventional response endpoints to determine whether chemokine engineering provides durable clinical benefit rather than simply increasing short-term tumor entry.
